# Formalin-Induced Fluorescence Reveals Cell Shape and Morphology in Biological Tissue Samples

**DOI:** 10.1371/journal.pone.0010391

**Published:** 2010-04-28

**Authors:** Ulrich Leischner, Anja Schierloh, Walter Zieglgänsberger, Hans-Ulrich Dodt

**Affiliations:** Max Planck Institute of Psychiatry, Munich, Germany; Universidade de Vigo, Spain

## Abstract

Ultramicroscopy is a powerful tool to reveal detailed three-dimensional structures of large microscopical objects. Using high magnification, we observed that formalin induces fluorescence more in extra-cellular space and stains cellular structures negatively, rendering cells as dark objects in front of a bright background. Here, we show this effect on a three-dimensional image stack of a hippocampus sample, focusing on the CA1 region. This method, called FIF-Ultramicroscopy, allows for the three-dimensional observation of cellular structures in various tissue types without complicated staining techniques.

## Introduction

Microscopical imaging of biological tissue samples regularly has to overcome the fact that cells and their components are transparent and colorless. A cell consists of lipid membranes and proteins, surrounded by water, and all these constituents are normally transparent. In order to visualize the shape of a cell or the arrangement of its components, we thus need to apply special techniques. One such technique is phase contrast microscopy, a technique that resolves differences in the refractive index of a cell by use of a transmission light illumination. Fluorescence microscopy is another way to investigate the shape of a cell. In this technique, a fluorescent molecule is attached to the cell and illuminated with a suitable excitation wavelength, causing it to fluoresce. Fluorescence microscopy is a very powerful technique, because the detected light is directly generated by the fluorescence tag. This allows for a precise localization of the origin of the fluorescence light.

Although fluorescence microscopy is a very good technique to resolve the position of a fluorophore, we are facing a different challenge when using this technique: we must attach a fluorophore to the position of interest. A lot of sophisticated techniques were developed for this, such as the generation of transgenic animals, where a special type of cell also expresses a fluorescent protein, making the cell fluorescent. Another technique is the use of specific fluorescent antibodies, binding to the structure of interest, e.g. receptor proteins in the cell membrane. Fluorescent antibodies are highly specific, but it is difficult to get them close to the structure of interest, as they are quite large and cannot diffuse through a biological tissue sample for long distances. We cannot use this technique for large biological samples. If we want to apply antibodies, we must first cut the sample in thin slices. Only in this case, antibodies can reach all positions of the sample.

If we want to image the biological structure in three dimensions, the optical situation is a bit more complicated, as we must additionally detect the depth-position of the fluorescent tag. The standard three-dimensional microscopical techniques (confocal and 2-photon) resolve the depth-position by using a focused beam of excitation light, as this mainly generates fluorescence at the focus spot. The three-dimensional structure is then resolved by scanning the object with the spot of focused light. In this case, the size of the focus spot determines the resolution. The resolution can be increased by focusing to a smaller spot, but this also increases light intensity. These high light intensities require a robust non-bleaching fluorescence signal. A lot of effort is directed at improving dyes or attaching a robust dye to an interesting structure. However, weak or fast fading fluorescent signals can not be resolved three-dimensionally with these techniques.

**Figure 1:**
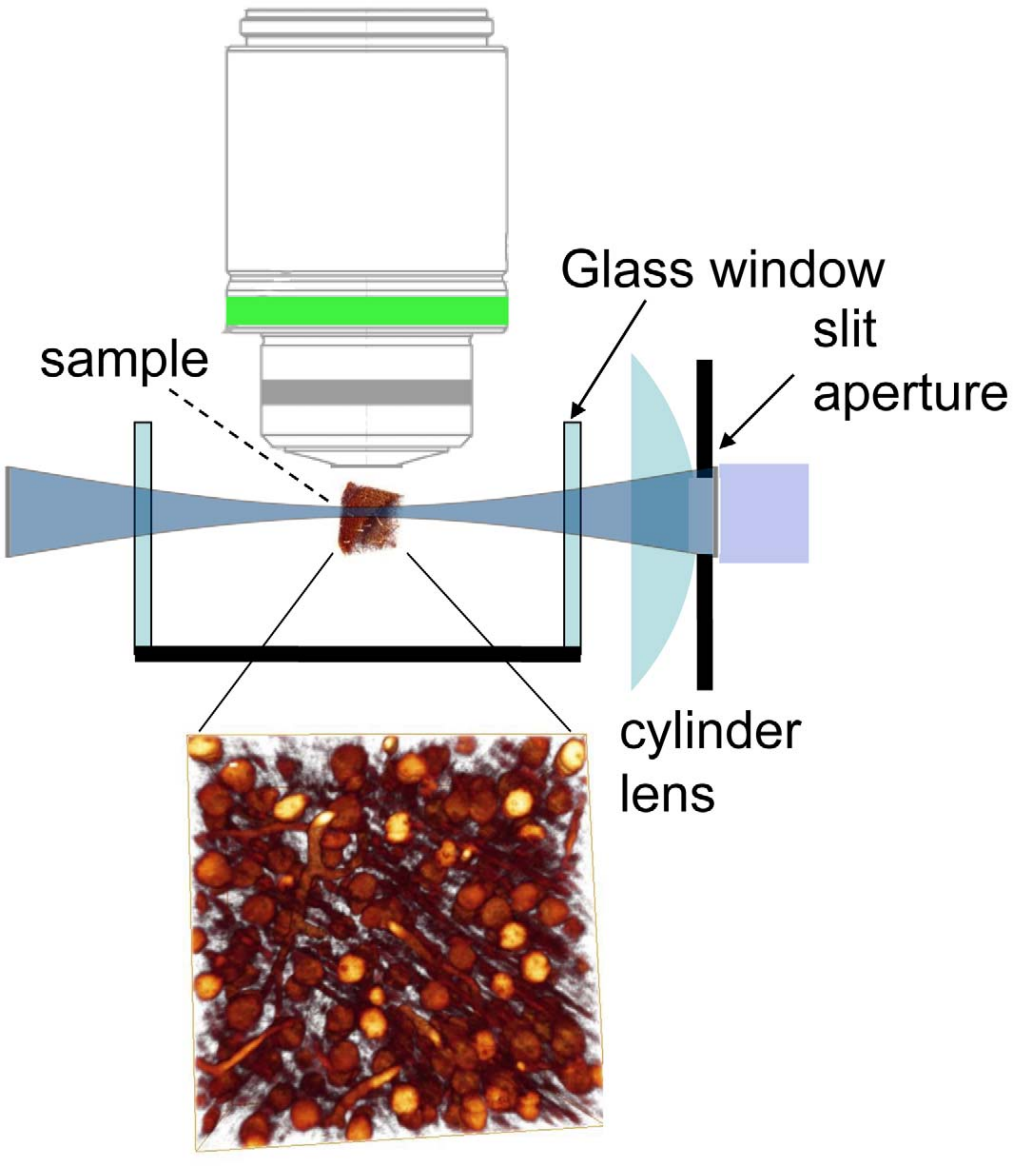
Principle of illumination of ultramicroscopy. We only illuminate the focus region from the side, hence other parts of the sample are not bleached. This allows for the detection of fast bleaching fluorescence signals.

Recent studies suggest an alternative illumination technique [1], [2], [3], [4] originally called Ultramicroscopy [5] (fig. 1). Instead of point-wise scanning, a planar illumination from the side is applied, avoiding high light intensities. Additionally the illuminating light does not penetrate the whole sample in z-direction. Only the focus region is illuminated, hence other parts of the sample are not bleached, as in confocal microscopy. This illumination technique avoids the restriction on strong and non-bleaching fluorescent dyes. We use this technique with an additional histological procedure that clears biological samples to complete transparency [6]. The main principle of establishing transparency is to replace the water of the biological sample with a liquid of the same refractive index, hence scattering effect are minimized and the sample becomes transparent. This histological technique allows for optical imaging in deep regions of the tissue sample [1]. This technique, in combination with Ultramicroscopy, allows for the acquisition of optical images of weak and fast fading fluorescence signals in deep tissue layers in three dimensions [1].

Another challenge in biological imaging is the conservation of the sample. Without oxygen supply, cells rapidly change their shape and finally trigger the apoptosis pathway, a mechanism of controlled ‘suicide’ of a cell. This mechanism results in a complete self-destruction, involving a disruption of the cell membrane and a disassembly of all parts of the cell. If we want to observe the cells in the natural condition, we must stop all metabolic activity as fast as possible by adding certain substances. The substances mainly used are formalin (the aqueous solution of formaldehyde), paraformaldehyde and sometimes glutaraldehyde. These molecules have highly reactive ends and cross-link the proteins and lipids. Metabolic activity is stopped immediately and the morphological structure is fixed after adding such a substance, but these substances have some side-effects. They can cover and change the surface of a cell, and a specific antibody can sometimes no longer recognize a certain molecule. Additionally, these substances generate some background fluorescence, normally referred to as artifact.

## Methods

**Figure 2:**
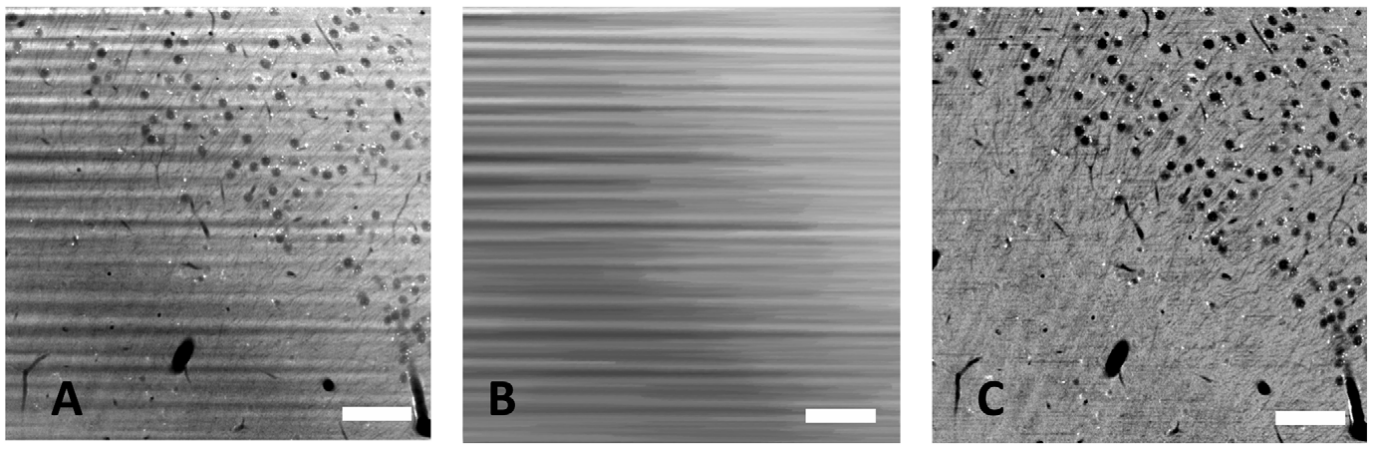
The stripe removal algorithm. A: Original image data from hippocampus region, blood vessels and nerve cells with dendrites are visible. The stripe-shaped illumination artifact is caused by shadow-producing impurities in the tissue sample. We illuminated from the right side. B: image after nonlinear horizontal smoothing, C: image after dividing image A by B. This stripe-removal algorithm corrects the non-homogeneous illumination. (all images are contrast adjusted, scale bars 50 microns).

The formalin-induced artifact fluorescence is very weak and fast fading, but it exhibits a surprising distribution: Extracellular areas contain more of this fluorescence than intracellular areas, rendering cells as dark objects on a bright background (fig. 2). Formalin is our standard substance for conservation of biological tissue samples, hence this signal was present in all our images with an extremely low intensity. By using the illumination technique of ultramicroscopy, together with a high-sensitivity camera and a long acquisition time, we revealed this signal in a clarity previously unseen. We call this method FIF-Ultramicroscopy, because we image the formalin-induced fluorescence with Ultramicroscopy. The difference in fluorescence intensity is very reliable and displays even smaller structures like dendrites. Blood vessels appear in the same way, as they contain no fluorescence at all. With higher magnification, a typical illumination artifact becomes apparent: The sample is not homogeneously transparent, and the intensity of the illumination light is fragmented in a striped way (fig. 2a). We assume this is caused by non-transparent particles producing shadows afterwards. It could also be a non-homogeneous refractive index, resulting in phase-shifts in the illumination beam, causing interferences. The direction of the stripes is always in the direction of illumination, in our case from the right side (fig. 2a). This is a typical artifact of ultramicroscopy, present in many of our images [1] and in data from other groups, e.g. [7]. Correcting this artifact is necessary for image improvement and subsequent data analysis.

**Figure 3:**
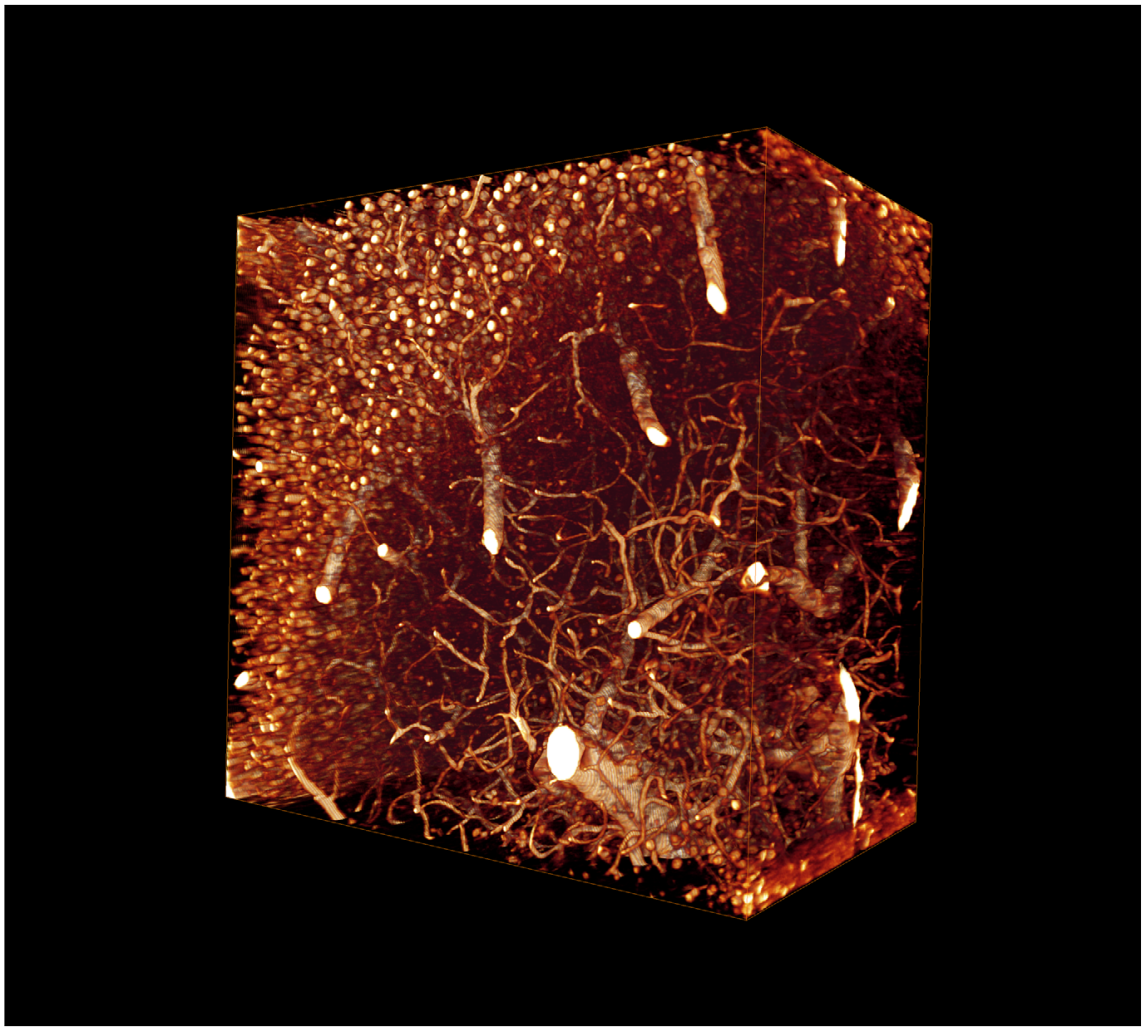
Three-dimensional display. View on the sample data (500×500×300 microns) of the hippocampus CA1 region, displayed with computer graphics. The section is about 0.5 mm under the surface of the excised hippocampus.

To correct this artifact, we applied a nonlinear smoothing operation based on mathematical morphology. As we know the orientation of the stripes we are able to smooth only in the direction of the stripes. The particular nonlinear smoothing operation we applied is a ‘closing’ followed by an ‘opening’, also named ‘rolling ball algorithm’ [8]. It removes all content from the images except the stripes. The smoothed image (fig. 2b) supplies the information of the local illumination of every pixel in the image. The final step is to divide pixelwise the original image (fig. 2a) by the smoothed image (fig. 2b). This corrects the non-homogeneous illumination, highlights the darker parts and darkens the brighter parts (fig. 2c). Finally we multiply all pixels with the average intensity to get the pixel values in the normal range again. This simple algorithm dramatically increases the image quality and clearly displays the original structure without artifacts. The images are now in a format that allows for 3d visualization and automated data analysis (fig. 3, Movie S1, Movie S2).

## Results and Discussion

The weak intrinsic signal of formalin-induced fluorescence can be used to reveal the cellular structure of tissue samples. This can be reliably detected with ultramicroscopy. This combination avoids the generation of transgenic animals with fluorescent cells or a complicated histological staining procedure. This technique is not limited to brain tissue, but can be applied to all tissues that do not contain pigments, as they absorb the illumination beam and the emitted fluorescence.

This 3D imaging technique can be used for various investigations. Trajectories of large objects like blood vessels can be tracked with high precision and provide important information about branching patterns of vessels in tumor genesis and metastasis formation. Number, size and distribution of cell bodies and dendrites within a given region of a brain can be determined, which is an important indicator of brain damage or development. Another structure of high interest in the brain are dendritic spines, small protrusions (about 1 micron) on the surface of nerve cells. Their position and number changes in the adult brain and can be influenced by a number of parameters, such as behavioral tasks that require memory formation. We are convinced that these can be revealed with a higher magnification.

The reaction of formalin with proteins can be characterized by cross-linking the proteins [9]. This can form chemical ring structures with an increased fluorescence. The formalin-induced fluorescence can be highly specific and was already used to visualize special nerve cell subtypes [10], [11], [12], especially to detect biogenic monoamines. This was already visible 30 years ago. The now increased sensitivity allows for a better measurement, and we can detect slight differences in the intrinsic fluorescence between extracellular and intracellular components. This effect was mainly visible with formalin. When using paraformaldehyde (PFA) instead of formalin, we could hardly see this signal by eye, but we were by far not able to record it with a camera. It was some magnitudes lower and could not be detected with the needed contrast. PFA is often used because it produces almost no intrinsic fluorescence. Glutaraldehyde is also a common substance for fixation, but it is not often used because it causes even higher background fluorescence than formalin. The use of Glutaraldehyde could also produce some interesting intrinsic signals.

The reason for the extracellular increased fluorescence could be a different protein composition of the extra-cellular matrix (ECM). The ECM can be highly complex and their composition also influences the cell. For example, in cancer research there are a lot of investigations on the local environment of a cell, and how the environment can influence the aggressiveness of a cell. The local environment of cells in metastasis is sometimes similar to a wound, as a wound requires fast growing cells for healing processes. An environment similar to a wound activates cellular pathways that enable cell divisions and growth, and drive the cancer cells to a much more aggressive behavior [13]. A lot or research is done to investigate the composition of the ECM and its protein constituents [14], especially in cancer research.

The resolution applied here is not sufficient to detect differences of intracellular organelles. As the formalin-induced fluorescence is somehow specific, there could be a difference of autofluorescence levels in different organelles. There might be some additional discoveries when this technique is applied with a higher resolution. Such effects could not be seen with this resolution. The xy-resolution is about 0.5 micron, the z-resolution about 5 microns [15]. Other cell types like glia cells are not visible. Maybe these cell types have a different composition of proteins that result in a different formalin-induced fluorescence.

**Figure 4:**
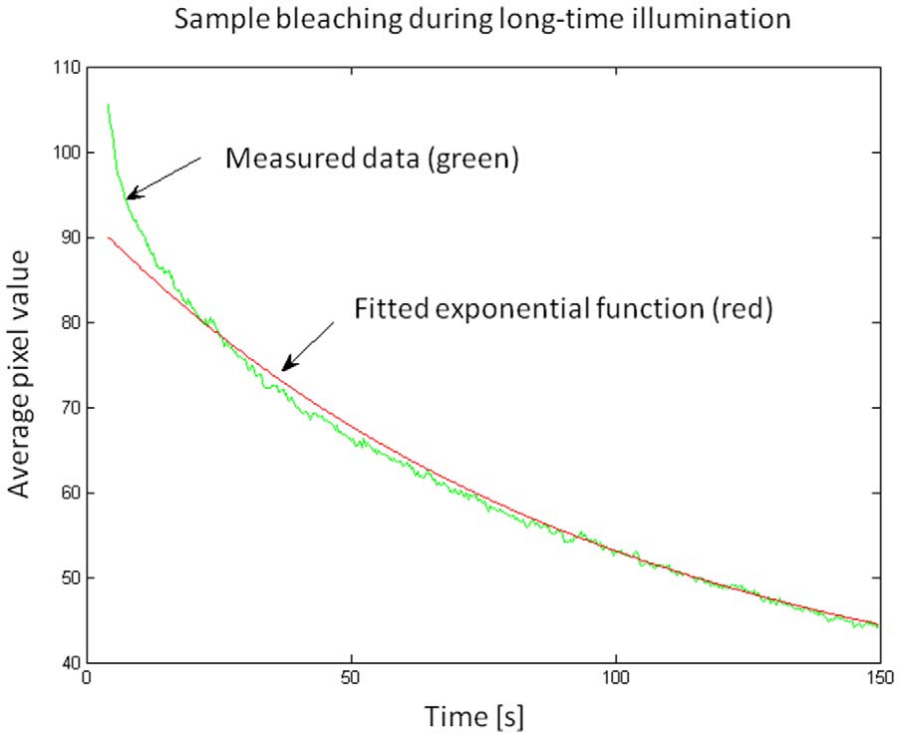
Sample bleaching experiment. The formalin-induced fluorescence bleaches relatively fast, but with ultramicroscopy it can be preserved long enough for recording.

The formalin-induced fluorescence bleaches quite fast and this process is critical for image acquisition. Therefore, we investigated the bleaching properties by illuminating the same position and recorded 3 images per second for more than 2 minutes with the standard acquisition parameters for illumination intensity (illumination intensity: 1–2 mW per mm slit length, 300ms acquisition time per image). The decline in fluorescence intensity is clearly visible (fig. 4). To analyze the decline of the fluorescence, we plotted the average pixel intensity versus the time. The function resembles to an exponential decay. To find the decay-constants we tried to fit an exponential function and found that a single exponential fit can not describe the bleaching very well, as there is a fast bleaching component that initially contributes about 20–30% of the absolute fluorescence, but fades out within the first 5–10 seconds, and a more stable one with a lifetime in the range of minutes. We interpret this as a hint that there are at least two different kinds of formalin-induced fluorophores.

The bleaching process is critical. Confocal microscopy illuminates through the objective lens and the illumination beam penetrates the whole sample in z-direction. This bleaches other positions of the sample above and below the focal plane as well. This effect increases with the number of imaging planes [16]. Ultramicroscopy avoids this out-of-focus illumination and this kind of bleaching process is completely absent. Although confocal microscopy should be able to image this signal for some images of a z-stack, it is by far not possible to record a z-stack of some thousands of images, like we routinely do.

The illumination technique of ultramicroscopy treats the sample with more care and dramatically reduces bleaching. This way, we can preserve the formalin-induced fluorescence long enough to record it with the needed precision. It is also possible to record the same sample multiple times. Multiple recordings are very promising, as the sample can be recorded from different orientations. These different data stacks can be used for image improvement [17]. As the depth-resolution is especially worse, about 5–10 times worse than the xy-resolution [15], an additional imaging of the sample rotated by 90° would allow us to map all dimensions with high precision [16].

Analysis of this three-dimensional image data requires automated methods. Blood vessels can be isolated by connectivity analysis, and region-growing algorithms are the method of choice for investigations on the morphology of individual cells. Interdisciplinary collaborations between theoretical sciences, like image processing and pattern classification on one side and life sciences like pharmacology and medicine on the other side, are necessary to use this technique to its fullest impact. It provides an easy access to answers to a variety of questions on the condition of a biological sample.

### Sample preparation and data gathering

Mice were perfused with isotonic phosphate-buffered saline to flush the vessels, and then with isotonic phosphate-buffered 4% formalin solution for fixation. It is important to remove all remaining blood during the perfusion from the blood vessel system, as remains of blood have a high fluorescence and results in a bright signal. We already used this property to display the blood vessel system in embryos [1]. Autofluorescence of blood is much brighter than the formalin-induced fluorescence. It is difficult to observe the formalin-induced fluorescence with the needed dynamic range when there is an additional bright object in the image. Perfusion must be done carefully and with low pressure to avoid a disruption of a blood vessel, as this would necessarily deposit some blood in the tissue. This property of blood will also limit the use of this technique in combination with experiments that require an insertion of a pipette in the brain, as such a procedure is normally accompanied with damages of the blood vessel system.

**Figure 5:**
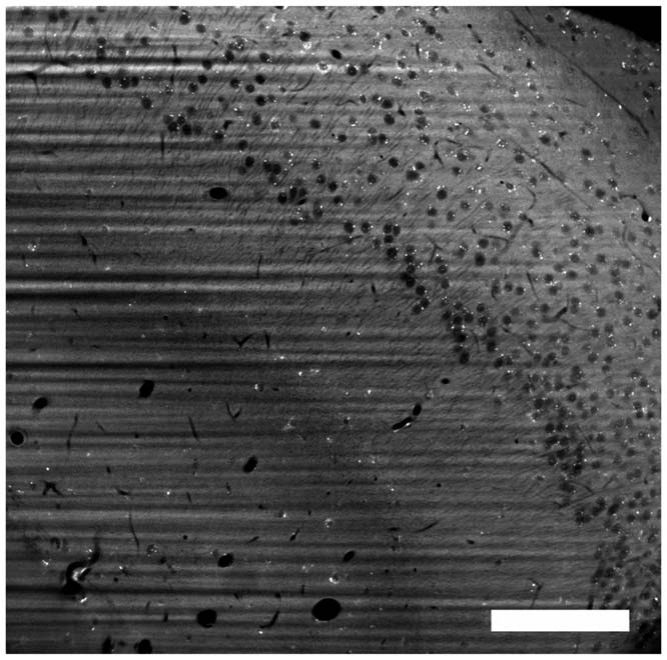
An original image from the z-stack. The image displays a worse contrast on the sides left and right, because the illumination beam is less focused there and illuminates a thicker part of the sample. We analyzed the beam shape and the depth-resolution with this illumination technique in a previous paper [15]. (scale bar: 100 micron).

In the next step after the perfusion, we isolated the brain, excised the hippocampi and cleared them with the Spalteholz-procedure [6]. We illuminated the sample with 488nm and a cylindrical lens (f = 40mm) and a slit aperture (d = 4mm) with 2 mW illumination intensity per mm slit length. This configuration results in a depth-resolution of 5µm and a field of sharp focus (the area where the illumination beam is thin enough to provide images of high quality) of 0.5 mm [15]. There is a slight decline of contrast at the edges of the image due to a thicker illumination light sheet (fig. 5). This is almost not visible in the 3d-animation, because the 3d display is done with a color-map with transparency-parameter. This resembles more of a threshold operation and the non-homogeneous contrast throughout the image is not visible. We recorded the emitted fluorescence (GFP-filter 505–555nm) with a 20× lens (Zeiss, LD, NA = 0.4). Image data was captured with a high sensitivity camera (Andor iXon 885, em-gain set to 48, an extreme signal amplification level) with an illumination time of 600ms. We moved the sample with a positioning-device by 0.5 µm in the z-direction between each image acquisition, hence scanning the whole sample in the depth-direction. Experimental procedures with mice were approved by the committee for the Care and Use of Laboratory Animals of the Government of Bavaria.

The selection of the right objective lens is of major importance for the image quality, especially for this application. The sample is immersed in a mixture of benzylbenzoate and benzylalcohol (2∶1) [6]. This liquid has a refractive index of 1.55, hence we need an objective lens for oil immersion. Additionally we need a working distance of at least 1–2 mm, as this is the size of the excised sample. Oil immersion objective lenses are normally used for highest resolution. High resolution objective lenses have high numerical apertures and low working distances (100–200µm), as they are developed for investigations of slice preparations. There are simply no objective lenses for our exotic application, that requires an optical correction for oil immersion and a long working distance. We ‘misused’ long-distance objective lenses from Zeiss and Olympus for inverted microscopes, developed for observations of cells at the bottom of a glass container through a thick wall of glass. These objective lenses have a correction collar for the glass thickness that allows for an optical compensation for the different refractive index of the glass. Because the refractive index of glass is about the same as of the immersion medium, we gained good results when the correction collar is adjusted to a value equal to the deepness of the observed section in the immersion medium. The products of the two possible vendors (Zeiss and Olympus) are of a slightly different characteristic: according to our subjective impression, the Zeiss objective lenses have a higher light transmission, but a worse image quality. Olympus is the opposite. This may be due to the choice of different types of glass. Therefore, the products from Zeiss are superior for lowest light applications. The disadvantage of the Zeiss products is the different correction optics. The working distance changes when we change the correction collar to a different value, hence we must refocus the microscope. Finding the right values is an iterative procedure and not easy. Additionally, the right values must be found relatively fast, as the sample bleaches quite fast and we need a strong signal with high contrast to determine the image quality and the settings of the correction collar. Finally, the cylindrical lens must be placed with sub-millimeter precision. All these adjustments are quite tricky and require an experienced operator of the microscope. A suited objective lens (oil immersion and long working distance) would make the acquisition of such high-resolution data much easier.

The three-dimensional animations were created with the program Amira (version 5.2.0). We display the preprocessed (without stripes), inverted and contrast adjusted data. We applied the volume-rendering module ‘voltex’ with the ‘volrenGlow’ colormap. This colormap is well suited for displaying three-dimensional structures, as the objects are rendered with a soft and natural surface.

### The stripe-removal algorithm

**Figure 6:**
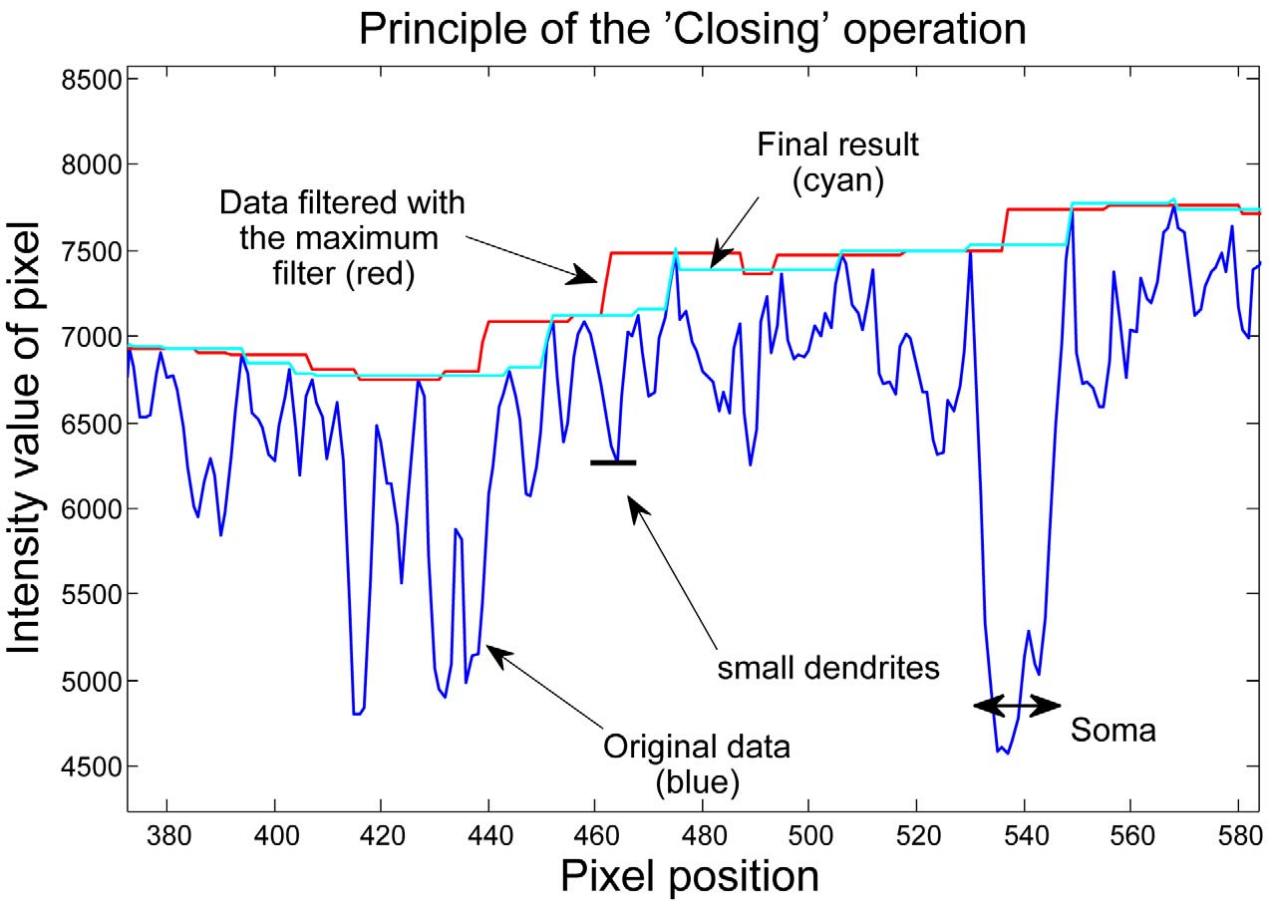
The ‘Closing’ operation. A maximum-filtering followed by a minimum-filtering closes all ‘holes’ in the intensity graph and smoothes the graph to the background level. The large holes are somata, small holes are dendrites.

We apply mainly functions from the framework of mathematical morphology. The nonlinear smoothing operation is composed of a Closing, followed by an Opening. The Closing operation is composed of two steps: a maximum filtering followed by a minimum filtering (fig. 6). In the first step, the maximum filtering, we assign every pixel a new pixel value, the maximum of the neighboring pixel values. The size of the neighborhood is the critical parameter. We, for example, choose a diameter of 25 pixel, 12 pixel on each side. The pixel in the middle gets the maximum pixel value of his 24 neighboring pixels. This operation fills up ‘holes’ depending of their size. If a ‘hole’ is smaller than 25 Pixel, it is being filled up at the level of the background intensity. As this operation also shifts edges, the following minimum-filtering reverses the edge-shifting. This operation smoothes all ‘holes’ in the intensity graph and fills them up at the background level and the name ‘Closing’ is intuitively clear. If a hole is larger than the filtering element, it is ignored and remains in the intensity graph. An Opening is the opposite operation, a minimum filtering followed by a maximum filtering. It removes small peaks from the intensity-graph. The Opening- and Closing-operations select objects of the intensity graph according to their lateral size, and independent of the offset- or background value. We apply an opening after the closing to remove small peaks that are caused by noise. This results in a smoother final graph.

Subsequent Opening- and Closing- operations are called the rolling ball operation [8], [18], as the resulting graph resembles the line where the surface of a ball is rolling over the intensity graph. With this operation, we can smooth the image to the background intensity and remove all content from the image. In contrast to the standard linear smoothing techniques, these morphological filters are independent of the absolute intensity values or the difference of brightness-values between a soma and background. They smooth everything with a lateral size smaller than the filter element, regardless of the brightness of the object.

The shape of the structuring element defines which content will be removed from the image. It must have a line-shape and the line must be oriented in the direction of the stripes. In this way, we selectively remove horizontal content only. As the structuring element is not extending vertically, it is blind for vertical changes and removes everything except the stripes. We interpreted the resulting image as the local illumination of every pixel. To obtain the final image, we pixelwise divided the original image by the illumination image and then multiplied every pixel with the average intensity of the smoothed image.

The final algorithm was refined a bit. There are a number of small highly fluorescent spots located at the surface of the somata. The origin of this specific intrinsic fluorescence is unknown, but it should not be overrated. It is just about three times higher than the formalin-induced fluorescence background. These bright spots cause problems in the initial closing. In the case when two of these highly fluorescent points are located horizontally close, the initial closing operation would fill the distance in-between on the high pixel value. This would results in a bright line in the illumination image (fig. 2b). To avoid this, we first isolate and remove the small bright spots by isolating small details using the TopHat filter [19] and a threshold-operation, selecting small details of extreme brightness. After this short preprocessing step, the rolling ball algorithm works quite fine.

The overall procedure works well as long as the structural element is larger than the details in the image. If a blood vessels or a dendrite is oriented in the direction of the stripes and it exceeds the structuring element, it will be considered as a horizontal line and will not be removed in the illumination image. It will be removed in the next step, because there we remove everything that was not removed by the smoothing operation before.

We can execute the stripe-removal algorithm with just three commands:

ImageWithoutPoints = OriginalImage-…

imtophat(OriginalImage,strel(’disk’,3))…

.*uint16(imtophat(OriginalImage,strel(’disk’,3))>1000);

IlluminationImage = …

imopen(imclose(ImageWithoutPoints,strel(’line’,25,angle)),…

strel(’line’,25,angle));

Result = uint16((double(ImageWithoutPoints)… ./double(IlluminationImage)).*5000);

The code is written in the programming language M (MatLab interpreter with image processing toolbox). Some parameters must be adjusted for each data type, e.g. the angle of the stripes or the size of the structuring element that initially removes the bright fluorescent points, but this is a very easy task for a person who is familiar with the programming environment and its syntax.

This technique also corrects the non-homogeneous illumination caused by absorption. In our image (fig. 2a) the right side is in general a bit brighter than the left side, as the illumination beam is more intense there. The first step detects the local illumination intensity. The second step then compensates the local non-homogenous illumination. These two steps also compensate the weaker illumination on the left side.

The framework of mathematical morphology provides a good toolbox for sorting the content of an image by its shape, size or orientation. The methods are fast and robust. A lot of work can be done using just these methods. Only when the objects are about the same size and the shape is not clearly defined (like small blood vessels versus cell bodies of pyramidal cells), more sophisticated methods from machine learning and artificial intelligence are necessary for automated component discrimination and image analysis.

## Supporting Information

Movie S1 Three-dimensional clipping-plane animation of the image data. Computer-generated three-dimensional animation of hippocampus CA1 tissue (image size: 500×500×300 microns), visualized with volume-rendering technique.
(9.94 MB WMV)

Movie S2 Three-dimensional rotation-animation of the image data. Computer-generated three-dimensional animation of hippocampus CA1 tissue (image size: 500×500×300 microns), visualized with volume-rendering technique.
(9.10 MB WMV)

## References

[1] Dodt H-U, Leischner U, Schierloh A, Jahrling N, Mauch CP (2007). Ultramicroscopy: three-dimensional visualization of neuronal networks in the whole mouse brain.. Nat Methods.

[2] Holekamp TF, Turaga D, Holy TE (2008). Fast three-dimensional fluorescence imaging of activity in neural populations by objective-coupled planar illumination microscopy.. Neuron.

[3] Huisken J, Swoger J, Del Bene F, Wittbrodt J, Stelzer EHK (2004). Optical sectioning deep inside live embryos by selective plane illumination microscopy.. Science.

[4] Voie A, Burns D, Spelman F (1993). Orthogonal-plane fluorescence optical sectioning: three-dimensional imaging of macroscopic biological specimens.. J Microsc-Oxford.

[5] Siedentopf H, Zsigmondy R (1903). Über Sichtbarmachung und Grössenbestimmung ultramikroskopischer Teilchen, mit besonderer Anwendung auf Goldrubingläser.. Ann Phys.

[6] Spalteholz W (1911). Über das Durchsichtigmachen von menschlichen und tierischen Präparaten.

[7] Buytaert JAN, Dirckx JJJ (2009). Tomographic imaging of macroscopic biomedical objects in high resolution and three dimensions using orthogonal-plane fluorescence optical sectioning.. Appl Optics.

[8] Sternberg SR (1986). Grayscale Morphology.. Comput Vision Graph.

[9] Fraenkel-Conrat H, Olcott HS (1948). Reaction of formaldehyde with proteins VI. cross-linking of amino groups with phenol, imidazole, or indole groups.. J Biol Chem.

[10] Corrodi H, Jonsson G (1967). The Formaldehyde Fluorescence Method For The Histochemical Demonstration Of Biogenic Monoamines A Review On The Methodology.. J Histochem Cytochem.

[11] Falck B, Hillarp N-A, Thieme G, Torp A (1962). Fluorescence of catechol amines and related compounds condensed with formaldehyde.. J Histochem Cytochem.

[12] Rost FWD, Rost FWD (1995). Induced Fluorescence.. Fluorescence Microscopy II.

[13] Chang HY, Sneddon JB, Alizadeh AA, Sood R, West RB (2004). Gene expression signature of fibroblast serum response predicts human cancer progression: similarities between tumors and wounds.. PLoS Biol.

[14] Ayad S, Boot-Handford R, Humphries M, Kadler K, Shuttleworth C (1998). The Extracellular Matrix—Facts Book.

[15] Leischner U, Zieglgänsberger W, Dodt H-U (2009). Resolution of Ultramicroscopy and Field of View Analysis.. PLoS ONE.

[16] Reynaud EG, Kržic U, Greger K, Stelzer EHK (2008). Light sheet-based fluorescence microscopy: more dimensions, more photons, and less photodamage.. HFSP J.

[17] Wein W, Blume M, Leischner U, Dodt H-U, Navab N (2007). Quality-based registration and reconstruction of optical tomography volumes.. Lecture Notes in Computer Science.

[18] Sternberg SR (1983). Biomedical Image Processing.. Computer.

[19] Gonzalez RC, Woods RE, Eddins SL (2009). Digital image processing using MATLAB. 2. ed.

